# Chronic development of collagen-induced arthritis is associated with arthritogenic antibodies against specific epitopes on type II collagen

**DOI:** 10.1186/ar1800

**Published:** 2005-07-27

**Authors:** Estelle Bajtner, Kutty S Nandakumar, Åke Engström, Rikard Holmdahl

**Affiliations:** 1 Medical Inflammation Research, Lund University, Lund, Sweden; 2 Uppsala Biomedical Center, IMBIM, Uppsala, Sweden

## Abstract

Antibodies against type II collagen (CII) are important in the development of collagen-induced arthritis (CIA) and possibly also in rheumatoid arthritis. We have determined the fine specificity and arthritogenicity of the antibody response to CII in chronic relapsing variants of CIA. Immunization with rat CII in B10.Q or B10.Q(BALB/c×B10.Q)F_2 _mice induces a chronic relapsing CIA. The antibody response to CII was determined by using triple-helical peptides of the major B cell epitopes. Each individual mouse had a unique epitope-specific response and this epitope predominance shifted distinctly during the course of the disease. In the B10.Q mice the antibodies specific for C1 and U1, and in the B10.Q(BALB/c×B10.Q)F_2 _mice the antibodies specific for C1, U1 and J1, correlated with the development of chronic arthritis. Injection of monoclonal antibodies against these epitopes induced relapses in chronic arthritic mice. The development of chronic relapsing arthritis, initially induced by CII immunization, is associated with an arthritogenic antibody response to certain CII epitopes.

## Introduction

Rheumatoid arthritis (RA) is an autoimmune disorder characterized by a chronic erosive inflammation in joints leading to the destruction of cartilage and bone. The mechanisms behind RA are still unclear but early therapy with disease-modifying drugs such as antibodies against tumor necrosis factor-α or methotrexate reduce disease manifestations, and treatment with anti-CD20 antibodies depleting B cells gives promising results [1]. The autoimmune targets in RA are not known but autoantibodies against various joint-related epitopes are detected in sera. Antibodies against epitopes modified by citrullination show the highest specificity for RA and can be detected very early in the disease course [2-4]. Antibodies against type II collagen (CII) occur in a subset of RA, and CII-specific B and T-cells have been identified in rheumatoid synovium and synovial fluid [5-10].

Immunization of mice with CII leads to the development of arthritis, the collagen-induced arthritis (CIA) model for RA. CII-specific activation of both T and B cells is critical for the development of arthritis, and the transfer of both rodent [11] and human [12] serum with CII-specific antibodies induces arthritis in mice. Monoclonal CII-specific autoantibodies bind cartilage *in vivo *and induce arthritis [13]; the injection of large amounts of several of such mAbs in cocktails induces severe arthritis [14,15]. Collagen-antibody-induced arthritis (CAIA) is an inflammation that is dependent on Fc receptor and complement, involving the infiltration of both neutrophils and macrophages [15-18].

The antibody response to CII is predominantly directed towards the conformational triple-helical structures. Immunization with CII α-chains (denatured CII) induces only a weak antibody response and is not arthritogenic [19]. Therefore identification of the relevant B cell epitopes required the construction of recombinant triple-helical proteins and synthetic triple-helical peptides [10,20]. The major epitopes were identified with the use of series of mAbs from both mice and rats [13,20-22]. Interestingly, antibodies against some of the major epitopes (C1 and J1) are arthritogenic, whereas antibodies against others (F4) are not [10]. The immunodominance of these epitopes seems to be shared between both CIA in mice and rats and in humans with RA [10,20,22,23].

Until now, CIA has mainly been studied as an acute disease. Because RA is chronic progressive and shows relapsing inflammatory destruction of cartilage, we wished to investigate the antibody response and B cell epitope specificity in chronic CIA models; that is, with an active joint inflammation later than 6 weeks after the onset. The advantages of following the antibody response over a longer period are that we can find possible associations between epitope specificities and the different phases of the disease and can also find epitope shifts during the course of the disease. We have observed previously that mice with C57Bl/10 backgrounds tend to get more chronic arthritis although they are initially relatively more resistant than DBA/1 mice, for example [24]. We therefore immunized B10.Q mice, which have an arthritis-susceptible Aq class II congenic fragment on the C57B1/10 background, with rat CII, and found that they develop a chronic relapsing disease. We have also recently defined another strain combination, an F_2 _cross between B10.Q and BALB/c, that give an even more pronounced development of chronic arthritis. It could be shown that the changes in epitope specificity occur during the course of the disease. Interestingly, the C1, U1 and J1 epitope-specific antibodies were associated with the development of severe and chronic arthritis. Single injections of antibodies of each of these epitopes induced a relapse in chronic arthritic mice.

## Materials and methods

### Mice

All animals were bred and kept in a climate-controlled environment (temperature and humidity) with cycles of 12 hours light/12 hours dark at the animal facility of Medical Inflammation Research, Lund University. Male B10.Q mice and B10.Q(BALB/c×B10.Q)F_2 _mice of both sexes (8 to 12 weeks old) were used for the CIA experiments. B10.Q(BALB/c×B10.Q)F_2 _mice (45 to 49 weeks old) were used for the induction of relapse experiment. Local animal welfare authorities permitted all the animal experiments.

### Induction and evaluation of CIA

B10.Q mice (*n *= 25) were immunized intradermally (i.d.) at the base of the tail with 100 μg of rat CII in 0.1 M acetic acid, emulsified in complete Freund's adjuvant (CFA; Difco, Detroit, IL, USA) [25]. They were boosted subcutaneously on day 35 with 50 μg of CII in incomplete Freund's adjuvant (IFA; Difco, Detroit, IL, USA). Control mice (*n *= 5) were immunized with 0.1 M acetic acid emulsified in CFA and boosted on day 35 with 0.1 M acetic acid in IFA. Clinical scoring was performed for 156 days as described previously [25]; in brief, each inflamed toe or knuckle scores one point, whereas an inflamed wrist or ankle scores five points, resulting in a maximum score of 15 (five toes plus five knuckles plus one wrist/ankle) for each paw and 60 points for each mouse. The mice were scored twice or three times a week, except during the first 25 days when they were scored once a week. Serial eye bleeding was performed over a period of 156 days, three times a week during the first 5 weeks and then twice a week for the remaining time. To perform retro-orbital bleeding, capillary tubes (75 mm KEBO-Lab) were used and 30 μl of sera were collected at each bleeding time point.

B10.Q(BALB/c×B10.Q)F_2 _mice were immunized with 100 μg of rat CII emulsified in IFA administered i.d. at the base of the tail on day 0 and boosted i.d. on day 35 with 50 μg of rat CII in IFA. The mice were scored for a minimum period of 207 days for arthritis development, with the same scoring protocol as described above.

### Triple-helical peptides

The triple-helical peptides were synthesized essentially as described by Grab and colleagues [26]. The strategy for synthesis was as a first step to produce the peptide NH_2_-Lys-Lys-Tyr(tBu)-Gly-resin, creating a handle with three amino groups used for synthesis of the strands of the triple helix in parallel and creating a covalent link between all three strands at the carboxy terminus. The procedure of Grab and colleagues [26] was used with the following modifications. All synthesis was performed in an ABI 431 peptide synthesizer (Foster City, CA, USA) operated with the FastMoc procedure. The synthesis was performed with a capping procedure, minimizing the presence of peptide material lacking amino acids within the sequence. The resin used for synthesis was a Fmoc-glycine-Wang resin. All Fmoc (fluoren-9-ylmethoxycarbonyl) amino acid derivatives were used as single amino acids. The removal of the temporary lysine side chain protection ivDde [1-(4,4-dimethyl-2,6-dioxo-cyclohexylidene)3-methyl-butyl] was performed by treatment of the peptide resin with 2% hydrazine in *N*,*N*-dimethylformamide for 3 min; the procedure was repeated three times. The peptide resin was washed with *N*,*N*-dimethylformamide after treatment with hydrazine.

After completion of the synthesis the peptide was removed from the resin and all side chain protection groups were removed by treatment with a cleavage cocktail containing 5% phenol, 2% 1,2-ethanedithiol, 5% methyl phenyl sulfide, 5% water and 84% trifluoroacetic acid. The peptide resin was treated for 2 hours at 22°C, after which the resin was filtered off and the peptides were precipitated and washed with diethyl ether. Peptides were used without further purification. A control of the synthetic peptides was obtained by the analysis of tryptic fragments by MALDI-TOF (matrix-assisted laser desorption ionization-time-of-flight) mass spectrometry (Kompakt IV instrument; Kratos, Manchester, UK.) Chemicals were of analytical or synthetic grade, amino acid derivatives were obtained from Alexis Biochemicals (Lausen, Switzerland), and Fmoc-Lys(ivDde)-OH and Wang resin were from Novabiochem (Laufelfingen, Switzerland).

### ELISA

Microtiter plates (Corning Costar Corp., Cambridge, MA, USA) were coated overnight with either CII (10 μg/ml in PBS), or with one of the CII-specific epitopes C1, T1, J1 or U1 at 4 μg/ml concentration in PBS at 4°C and blocked with 1% bovine serum albumin (Sigma) in PBS for 1 hour at room temperature. The CII-specific response or the epitope specificities were determined by adding the sera at a standard dilution of 1:100 in PBS to the various CII-coated or epitope-coated plates for 2 hours at room temperature. Antibody binding was detected with horseradish peroxidase-conjugated goat anti-mouse total IgG (Jackson ImmunoResearch Laboratories, West Grove, PA, USA) and 2,2-azino-di-(3-ethylbenzthiazoline sulfonate) diammonium salt as substrate (ABTS tablets; Boehringer-Mannheim, Germany). The absorbance value was determined at 405 nm in duplicates. To determine the concentration of specific antibodies, we used a positive DBA/1 standard sera (titrated 0.5 to 10 μg/ml) and the computer program SOFTmaxPRO version 2.6.1. As standards for the epitope-specific ELISAs we used the mAbs listed in Table 1. In a similar manner to the polyclonal standard we calculated the amount of antibodies. However, because the monoclonal standards differ in affinity, the values are not comparable to each other and are therefore transformed to arbitrary units (AU). One AU is 1 μg/ml as calculated with a mAb standard. Normal B10.Q or B10.Q(BALB/c×B10.Q)F_2 _mouse sera were included in all assays and this absorbance value (0.1) determined the cut-off value.

### B cell hybridomas producing mAbs

The CIIC1, M2139 and 122.9 hybridomas have been described previously [13,21,22]. The UL1 hybridoma was established by immunizing a B10.Q mouse with 100 μg of rat CII emulsified in CFA. The mouse was first boosted subcutaneously at day 35 with 50 μg of rat CII in IFA. Five days before the fusion (day 210), the mouse was boosted a second time with 50 μg of triple-helical peptide containing the U1 epitope, in IFA, in the footpad. Draining lymph node cells were isolated and fused with myeloma (NSO) cells, and the subsequent cloning and anti-CII antibody selection were performed essentially as described previously [27]. For the production of antibodies the hybridomas were expanded in DMEM Glutamax-1 medium containing 0.5% streptomycin and 0.6% penicillin and Ultra low IgG fetal bovine serum (FBS) (Gibco BRL, Grand Island, NY, USA). The mAbs were purified from culture supernatants by affinity chromatography on protein G (GammaBind plus Sepharose; Pharmacia, Uppsala, Sweden) and all solutions for affinity chromatography were prepared in accordance with the GammaBind Sepharose manual. The mAbs were dialyzed against PBS. The concentrations of the mAbs were determined by freeze-drying. The solutions with antibodies were filter-sterilized with 0.2 μm syringe filters (Dynaagard, Spectrum Laboratories, CA) and stored at -70°C until used.

### Induction of relapses with mAbs in chronic mice

The B10.Q(BALB/c×B10.Q)F_2 _mice were immunized with 100 μg of rat CII emulsified in IFA i.d. at the base of the tail on day 0 and boosted on day 35 i.d. with 50 μg of rat CII in IFA. The mice were scored for a minimum period of 207 days for arthritis development. Mice that developed chronic arthritis (mice with severe arthritis for a minimum period of 120 days were considered to be chronic) were selected for this experiment. Clinical scoring was performed as described previously [25]. Groups of chronic mice (*n *= 6) were injected intravenously with a single mAb, 9 mg of either CIIC1, M2139 or UL1. Development of clinical arthritis was followed through daily visual scoring of the mice, starting the day after the antibody transfer and continuing until the end of the experiment. Arthritis was evaluated as described above.

### Statistical analysis

The Statview software program was used for the statistical analysis. The Mann-Whitney *U *(MW) test was applied to evaluate comparisons of antibody titres and scoring. The Pearson correlation coefficient, *r*, was calculated between the mean arthritis score and the logarithmic antibody response at specific time points. The *r *value measures the degree of relationship between two variables and varies between 0 and 1, where high *r *indicates a strong linear relationship. *P *< 0.05 was considered significant in all analyses.

## Results

### Chronic arthritis in B10.Q mice

**Figure 1:**
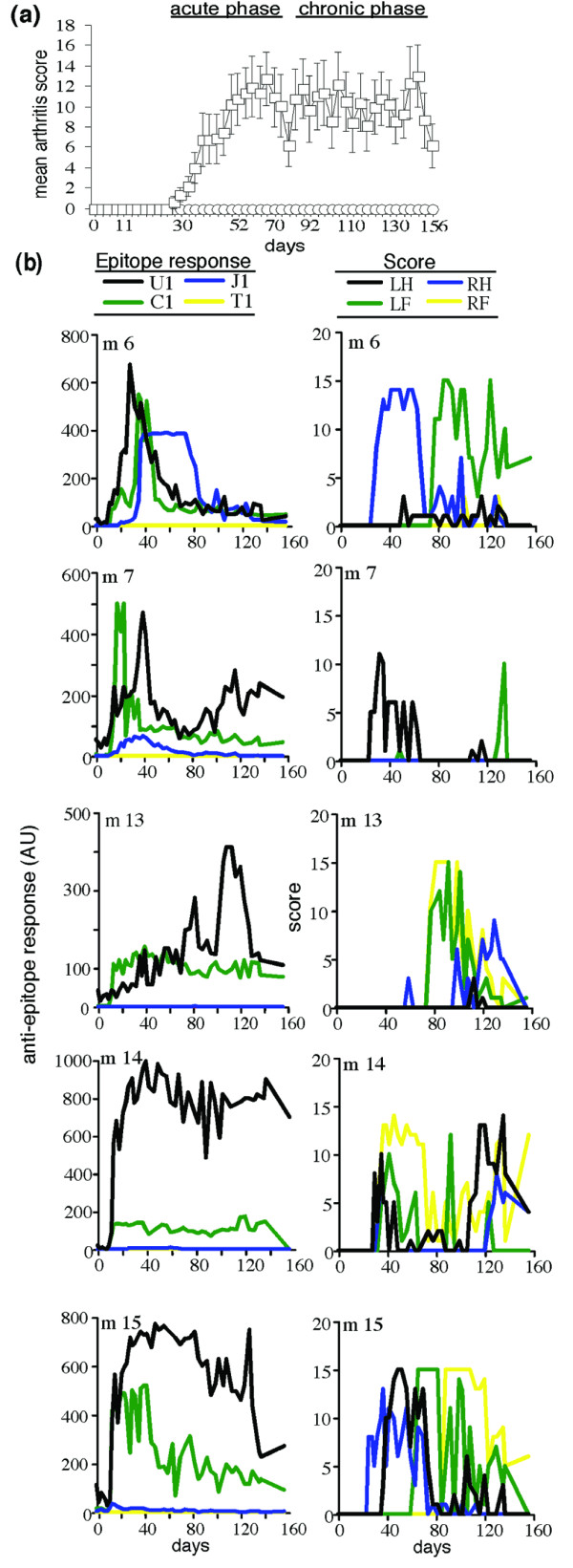
Arthritis development in B10.Q mice and antibody response to CII-specific epitopes in individual mice. **(a) **Arthritis course during 156 days of male B10.Q mice 8 to 12 weeks old (*n *= 25) immunized with CII in complete Freund's adjuvant on day 0 and boosted on day 35 with CII in incomplete Freund's adjuvant, showing the arthritis score of the diseased (*n *= 15, open squares) and healthy (*n *= 10, open circles) mice. The disease course is divided into an acute phase (days 0 to 70) and a chronic phase (days 74 to 156). The incidence was 60%. Results are means ± SEM. **(b**) Antibody response to specific CII epitopes and arthritis development in individual B10.Q mice. The epitope specificity in mouse 6 during the course of the disease shows the immunodominance of antibodies against the epitopes U1, C1 and J1. Arthritis disease course in mouse 6 indicates the score of the individual paws. LH is the left hind paw, RH is the right hind paw, LF is the left front paw and RF is the right front paw. The epitope specificity in mouse 7 during the course of the disease shows the immunodominance of antibodies against the epitopes U1 and C1. The disease course in mouse 7 indicates the score of the individual paws during the disease course. The epitope specificity in mouse 13 during the course of the disease shows the immunodominance of U1-specific antibodies. The disease course correlates with the U1 antibody response in mouse 13. The epitope specificity in mice 14 and 15 during the course of the disease shows the immunodominance of antibodies against the epitope U1. Antibodies against the C1 epitope were detectable in mouse 14 and were detectable before the onset of the disease and also during the acute phase of arthritis in mouse 15. The disease course in mice 14 and 15 indicates the score of the individual paws during the disease course.

To investigate the epitope specificity of B cells in sera from arthritic mice, we immunized male B10.Q mice (*n *= 25) to induce CIA. The mice started to develop arthritis on day 25 and reached an incidence of 60% (Fig. 1a). The disease course could be divided into two phases, acute (from days 0 to 70) and chronic (from days 74 to 156), because there was a decrease in the mean arthritis score on day 70 after immunization; that is, about 6 weeks after onset. Between these two phases there was a recovering period, during which the mean score value decreased. In the later phase, the paws were destroyed but clear relapses of arthritis could be detected in specific joints and during limited times, most clearly seen when the arthritis disease course is depicted for individual mice (Fig. 1b, mice 6, 7, 13, 14 and 15). We therefore defined the durations of the acute and chronic phases of the disease as above.

### The anti-CII response to specific epitopes in individual mice is immunodominant but shift during the disease course

For a specific screening of the sera against the major epitopes, we synthesized triple-helical peptides, covalently linked at the C terminus and with several glycine-proline-hydroxyproline triplets at the N terminus. The specificities of the peptides were shown by using mAbs for the various epitopes (Table 1). Analysis of the results of the individual sera clearly shows that each mouse had its own immunodominance pattern; that is, it responded mainly to one or a few epitopes. In addition, this response often sharply shifted during the course of the disease, possibly reflecting a clonal origin of this epitope spreading of the antibody response, see for example mouse 6 (Fig. 1b).

To be able to investigate the results from the anti-CII response to specific epitopes in individual mice and compare with the scoring during the same time period, we analyzed each paw in individual mice and found clear relapses of the disease (Fig. 1b). An example is mouse 7, in which the C1-specific antibodies dominated before arthritis, whereas U1 antibodies dominated during both the active acute phase and the active chronic phase of arthritis (Fig. 1b). In mouse 13, again the antibodies specific for U1 and C1 were immunodominant, but the U1 antibodies showed a very high response in the chronic phase and this correlated very well with the scoring curve for mouse 13 (Fig. 1b). In mouse 14, U1 antibodies dominated during both the active acute phase and the active chronic phase of arthritis, with very high antibody responses during the disease course (Fig. 1b). In mouse 15, the U1-specific antibodies dominated during the whole disease course (Fig. 1b). We could detect the C1-specific antibodies early on day 11 before we could see any clinical signs in this mouse. These antibodies dominated in the active acute phase of arthritis and then decreased.

These individual patterns of antibody responses indicate a strong environmental or stochastic factor in the selection of the antibody response to specific CII epitopes. However, links to the development of chronic relapsing arthritis are also indicated.

### The total anti-CII response

**Figure 2:**
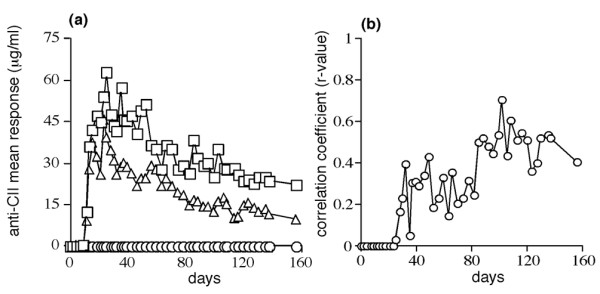
Development of antibody response to CII and correlation between arthritis severity and anti-CII response. **(a) **Mean antibody responses measured in sera against CII over time in control (open circles, *n *= 5), healthy (open triangles, *n *= 10) and arthritic (open squares, *n *= 15) mice. **(b) **Analysis of the correlation between the mean arthritis score and the anti-CII response during the disease course. Each point represents the correlation coefficient, *r*. The values of *r *are significant from day 85 to day 156.

Antibodies against CII were detected from day 11 and showed a sharp increase during the following days, but arthritis developed several weeks later (Fig. 2a). To investigate which role the CII-specific antibodies might have in the induction and perpetuation of arthritis during the course of the disease, we calculated correlations between arthritic scores and anti-CII antibody levels (Fig. 2b). Here we show that the correlation, *r*, between the arthritic score and anti-CII antibody levels increased with time and again confirm that animals developing arthritis had significant levels of anti-CII antibodies. To investigate whether the levels of anti-CII antibodies were more critical at the onset of the disease we compared the total anti-CII serum levels at 3 days before the onset day of each animal that developed disease with the levels in those that remained healthy; we found a more pronounced difference (*P *= 0.0002). Still, the total anti-CII antibody levels were polyclonal; to address whether epitope specificity might have a role we investigated the response against the major CII epitopes (denoted C1, J1, U1 and T1).

### Antibody responses against triple-helical peptides correlate with the late phase of arthritis

**Figure 3:**
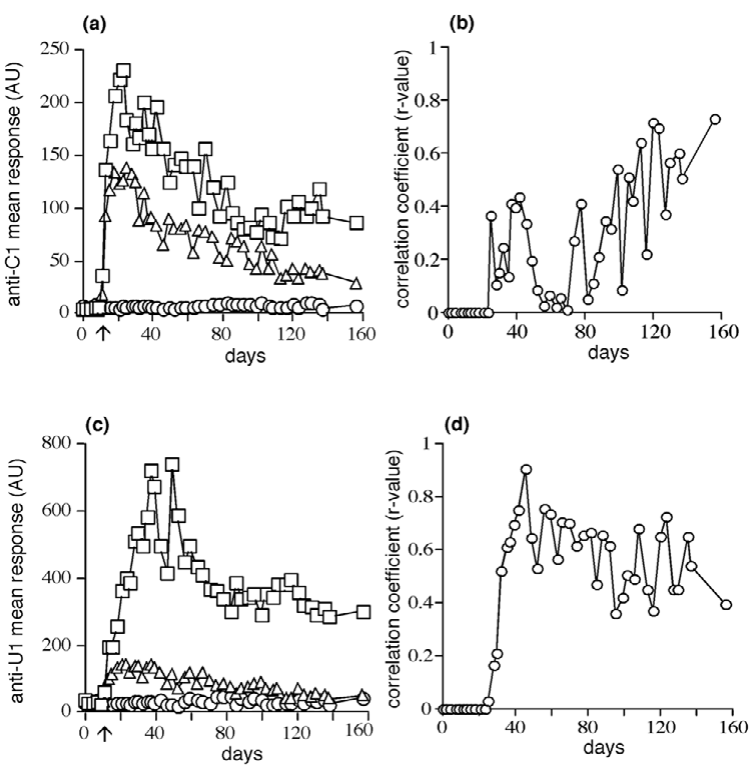
Antibody response to C1 and U1 and correlations between arthritis severity and anti-epitope response. **(a) **Mean antibody responses measured in sera against the C1 triple-helical peptide over time in control (open circles, *n *= 5), healthy (open triangles, *n *= 10) and arthritic (open squares, *n *= 15) mice. **(b) **Analysis of the correlation between the mean arthritis score and the anti-C1 response during the disease course. Each point represents the correlation coefficient, *r*. The values of *r *are significant on days 37, 42, 78, 92, 106 to 113 and 120 to 156 and range from *P *≤ 0.05 to *P *≤ 0.0001. **(c) **Mean antibody responses measured in sera against the U1 triple-helical peptide over time in control (open circles, *n *= 5), healthy (open triangles, *n *= 10) and arthritic (open squares, *n *= 15) mice. **(d) **Analysis of the correlation between the mean arthritis score and the anti-U1 response during the disease course. Each point represents the correlation coefficient, *r*. The values of *r *are significant on days 32 to 92, 99 to 113 and 120 to 156.

The antibody responses against the triple-helical peptides were measured by ELISA; we found that the mean antibody response against the triple-helical peptides varied. However, the antibody responses against C1 and U1 showed increased levels of antibodies as early as day 11 (Fig. 3a, c), whereas the levels of antibodies against J1 and T1 gradually increased to reach a maximal level at the onset of arthritis (data not shown). To investigate the possible role of the antibodies specific for C1 and U1 in arthritis during the course of the disease, we calculated correlations between arthritis scores and anti-epitope antibody levels (Fig. 3b, d). Here we show that the correlation, *r*, between the arthritic score and the anti-U1 antibody level is higher, with significant values of *r *for longer periods at different time points during the course of the disease than the correlation between the arthritic score and anti-C1 antibody level. To investigate these findings in another chronic arthritis mouse model, we analysed sera from a CIA experiment in B10.Q(BALB/c×B10.Q)F_2 _mice in the same way.

### B cell epitope specificity in a chronic relapsing arthritis model

**Figure 4:**
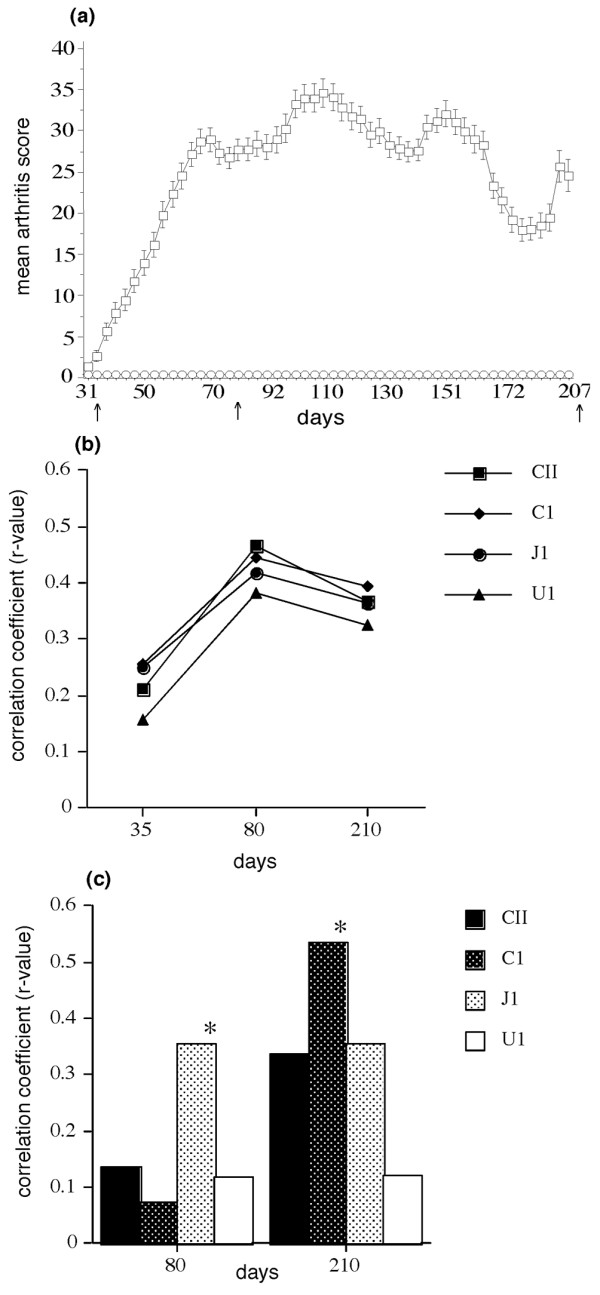
Arthritis development in B10.Q(BALB/c×B10.Q)F_2 _mice and correlations between relapsing arthritis and responses to CII/CII-specific epitopes. **(a) **Arthritis course during 207 days of male and female B10.Q(BALB/c×B10.Q)F_2 _mice (8 to 12 weeks old) immunized with 100 μg of rat CII emulsified in incomplete Freund's adjuvant (IFA) on day 0 and boosted on day 35 with 50 μg of rat CII in IFA, showing the arthritis score of the diseased (*n *= 96, open squares) and healthy (*n *= 94, open circles) mice. Arrows indicate the bleeding time-points (days 35, 80 and 210). Results are means ± SEM. **(b) **Analysis of the correlation between the mean arthritis score and the anti-CII or anti-epitope responses during the disease course. Each point represents the correlation coefficient, *r*. The values of *r *are significant on day 35, 80 and 210 for CII, C1, J1 and U1. **(c) **Analysis of the correlation between the mean arthritis score and the anti-CII or anti-epitope responses related to mice with a relapsing disease, either on day 80 (*n *= 32) or on day 210 (*n *= 21). Each bar represents the correlation coefficient, *r*. The values of *r *are significant for the J1 epitope on day 80 and for the C1 epitope on day 210.

To study the B cell epitope specificity in sera from another chronic arthritis model in mice, we investigated sera from B10.Q (BALB/c×B10.Q)F_2 _mice (*n *= 190) immunized for the induction of CIA. The mice were scored for a minimum of 210 days for arthritis development. About 4 weeks (on day 31) after the immunization, the mice started to develop a chronic relapsing disease (Fig. 4a). The incidence was 51%. The mice were bled on days 35, 80 and 210. We found a correlation between antibodies against CII, C1, J1 and U1 and the arthritic score on days 35, 80 and 210 (Fig. 4b). Although the values of *r *were low for CII and all the epitopes screened on day 35, they were significant. Because these mice showed a chronic relapsing disease, we further investigated the epitope specificity around the relapses.

### CII epitope-specific responses differ during different phases of arthritis progression

We analyzed sera taken on days 80 and 210 from mice that showed a relapse at about day 80 (ranging from days 75 to 85; *n *= 32) or at about day 210 (ranging from days 200 to 210; *n *= 21) for their epitope specificity. To investigate the role of the epitope-specific antibodies for the induction of the relapse, we made correlation studies between arthritic score and antibody level. Regression analysis showed a significant correlation between arthritic score and the antibody response against J1 epitope on day 80 and a significant correlation between arthritic score and the antibody response against C1 epitope on day 210 (Fig. 4c).

### Single anti-CII antibody induces relapses

**Figure 5:**
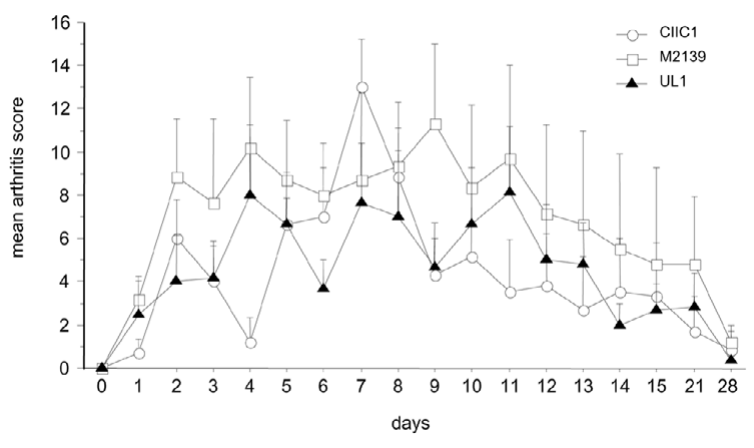
A single anti-CII antibody induces relapses. Induction of relapses in chronic mice (*n *= 6) injected with a single mAb, namely 9 mg of CIIC1 (open circles), M2139 (open squares) or UL1 (filled triangles). The incidence in the groups of mice injected with CIIC1 and M2139 was 100% and the incidence in the group of mice injected with UL1 was 83%. The selected chronic B10.Q (BALB/c×B10.Q)F_2 _mice had previously been immunized with 100 μg of rat CII in incomplete Freund's adjuvant (IFA) on day 0 and boosted on day 35 with 50 μg of rat CII in IFA. Mice that developed chronic arthritis (mice with severe arthritis for a minimum period of 120 days were considered to have chronic arthritis) were selected for the induction of relapses. Antibodies were injected on day 265 in these mice. Most of the mice had active arthritis before antibody injection (mean maximum score 8.1 ± 2.0). The basal level score from day 265 was subtracted from the observed arthritis score values in individual mice; 17% of these mice were relapsing on day 265. The basal levels of CII epitope-specific antibodies in sera just before injection of anti-CII antibodies are as follows: RCII, 95 μg/ml; C1, 15 AU; J1, 70 AU; and U1, 15 AU. Results are means ± SEM. AU, arbitrary units.

We wished to study whether a single anti-CII antibody is capable of inducing a relapse in mice with chronic arthritis. For this experiment we chose the chronic relapsing variant of CIA that develops in mice with mixed B10 and BALB/c backgrounds. B10.Q × (BALB/c×B10.Q)F_2 _mice were immunized with CII in IFA on days 0 and 35 and evaluated for arthritis for 210 days. These mice are genetically heterogeneous and only mice with severe and active chronic relapsing arthritis for a minimum of 120 days were selected for the experiment. The single injection of the antibodies CIIC1, M2139 and UL1 was performed on day 265 in these mice. Before antibody injection most of the mice had active arthritis (mean maximum score 8.1 ± 2.0). The basal level score from day 265 was subtracted from the observed arthritis score values in individual mice; 17% of these mice were relapsing on day 265. We injected 9 mg of single mAbs (CIIC1, M2139 or UL1). After injection, 80 to 100% of these mice were relapsing within 2 days (Fig. 5).

## Discussion

To study the role of a specific and arthritogenic B cell response to cartilage during a chronic relapsing arthritis disease course, we studied unique models of chronic relapsing CIA in mice. The development of chronic arthritis is related to an antibody response to defined epitopes on CII. Interestingly, the antibodies directed against distinct epitopes on the triple-helical part of CII correlate with chronic arthritis and induce arthritis relapses.

In the CIA model, the triggering of autoreactive B cells to CII is undoubtedly an important pathogenic factor during the acute phase of the disease. B cell-deficient mice are completely resistant to CIA [28] and the arthritis can be passively transferred with immune sera [11] and also induced with mAbs against CII [13-15]. However, the development of arthritis has not been perfectly correlated with serum titers of antibodies against CII because high titers of antibodies do not always lead to severe arthritis [29]. This is also observed in inbred strains, also suggesting other than genetic explanations. Thus, immunization of inbred mouse strains susceptible to CIA induces a variable titer of antibodies. In the present investigation evidence is presented for both a genetic and a non-genetic influence on the autoantibody specificity to CII. The analysis of chronic arthritis in B10.Q mice shows that individual but genetically identical mice varied markedly in specificity. However, mean values showed that the antibody response to mainly U1 and C1 were associated with the developments of chronic arthritis. The comparison with the genetically segregating F_2 _mice, in which BALB/c genes had been introduced showed that the individual variety led to a strong association to all of the investigated CII epitopes, the C1, U1 and J1 epitopes.

Chronic development of CIA has not previously been extensively investigated. In both the present study and one published previously [24] the C57Bl/10 background was used. In two of these experiments the involvement of C3H and BALB/c genes apparently promoted the development of chronicity. The development of chronic arthritis in the B10.Q strain was more pronounced than we normally observed; we assume that the boosting effect of repetitive bleeding could have enhanced the relapsing pattern.

The development of a unique epitope-specific antibody response shifting along the chronic disease course in each individual mouse is not only genetically controlled but also a stochastic process. An explanation could be that the selection of which B cell will form the germinal centers occurs as a response to CII derived from cartilage and that this selection is more or less random. The B cell response to CII is a strictly T cell-dependent process engaging germinal-center B cells that predominantly switch isotype to IgG and that to a large extent are somatically mutated [30,31]. Thus, for the new priming events to occur it is likely that both T and B cells specific for cartilage-derived CII are involved. Clearly, the specificity of the antibody response is changed during the development of chronic arthritis, indicating that the recognition of the CII used for immunization is different from that of CII derived endogenously from cartilage.

In the present experiments, in which we used only mice expressing the MHC class II molecule Aq, it is likely that the same B and T cell epitopes are recognized. A difference could be that the affinity for the T cell-recognized peptide in rat CII, used for immunization, is higher than in mouse CII [32]. Other contributing reasons could be that CII is exposed differently when scavenged from cartilage compared with when it is exposed in the immune inocula in the skin. The quality of the endogenous priming of the immune system is also likely to be different because neo-epitopes on CII in cartilage are formed by a changed glycosylation pattern [33,34] but also by processes modified by the inflammatory process itself, such as citrullination and oxidation [3,35,36]. The major epitopes on triple-helical CII, including C1, J1 and U1, seem to share a common motif, consisting of arginine-glycine-hydrophobic amino acid, which is possibly also located in a repetitive way on the cartilage surface or in CII aggregates, known to occur in an inflamed synovium [20,37,38].

As regards the consequence of the recognition of the changed epitope by antibodies we have now shown that antibodies against each of the major B cell epitopes on CII induce a relapse of arthritis in chronic arthritic mice. In most earlier studies the induction of collagen-antibody-induced arthritis requires both several anti-CII antibodies and a boosting injection of lipopolysaccharide [14,15,39]. As this was not necessary for inducing a relapse in chronic arthritic mice it is likely that the threshold for developing antibody-mediated arthritis in these mice is lower, possibly because they already have an ongoing anti-CII immunity. Nevertheless, it clearly shows that a sudden increase in antibody response to a single CII epitope, as we have noted to occur spontaneously during the course of the disease, is capable of inducing severe arthritis. The question arises whether this phenomena is unique to the mouse, whether we can use the mouse as a model to study the process in principle or whether in fact the same epitopes are engaged in other species, including humans. Interestingly, the data accumulated so far indicate that the epitope specificity of the response is shared between species. The major epitopes dominating so far are predominantly seen in mice and rats with CIA as well as in humans with RA [10,20,22,23]. Interestingly, antibodies against both the C1 and the U1 epitopes are positively correlated with RA, whereas the response to epitopes recognized by arthritis-protective antibodies recognizing the F4 epitope is not [10]. Even though RA is not primed by a CII immunization as in mice and rats, adaptive immunity to CII does occur in some patients, possibly because of exposure to modified CII from cartilage [40]. This could have a role in maintaining chronic inflammation in some cases of RA.

## Conclusion

We demonstrated that antibodies directed to distinct epitopes on the triple-helical part of CII are correlated with chronic arthritis and are arthritogenic in the mouse CIA model. We could show that each mouse develops a unique epitope-specific antibody response that may shift and spread to other epitopes during the course of the disease, indicating that new priming events occur. Clearly, the specificity of the antibody response is changed during the development of chronic arthritis, in particular engaging the major epitopes C1, U1 and J1. Injection of mAbs against these epitopes induces relapses in chronic arthritic mice. Thus, the development of chronic relapsing arthritis in mice, initially induced by CII immunization, is associated with an arthritogenic antibody response to certain CII epitopes.

## Abbreviations

CFA = complete Freund's adjuvant; CIA = collagen-induced arthritis; CII = type II collagen; DMEM = Dulbecco's modified Eagle's medium; ELISA = enzyme-linked immunosorbent assay; i.d. = intradermally; IFA = incomplete Freund's adjuvant; mAb = monoclonal antibody; PBS = phosphate-buffered saline; RA = rheumatoid arthritis.

## Competing interests

The author(s) declare that they have no competing interests.

## Authors' contributions

EB and KSN performed most of the experimental work and participated in preparing the manuscript. ÅE performed the synthesis of triple-helical peptide and helped in writing the manuscript. RH supervised, participated in the design of the study and helped in writing the manuscript. All authors read and approved the final manuscript.

## Figures and Tables

**Table 1:** Epitope sequences and epitope specificities of mAbs

Epitope	Epitope sequence (epitope in THP)^a^	MAbs		Antigen reactivity^b^				
		
		Clone	Isotype	CII	C1	J1	T1	U1
C1	GPBGPBGPBGPBGPBG-*ARGLTGRBGDA-*GPBGPBG-εACA	CIIC1	IgG2a	1.7	2.5	0.0	0.0	0.0
J1	GPBGPBGPBGPBGPBG-*MBGERGAAGIAGPK*-GPBGPBG-εACA	M2139	IgG2b	2.4	0.0	2.5	0.0	0.0
T1	GPBGPBGPBGPBGPBG-*IAGFKGEQGPKGEP-*GPBGPBG-εACA	122.9	IgG2a	1.7	0.0	0.0	2.5	0.0
U1	GPBGPBGPBGPBGPBG-*LVGPRGERGFB*-GPBGPBG-εACA	UL1	IgG2b	2.1	0.0	0.0	0.0	2.4

^a^All epitopes were in native triple-helical form. Amino acids are abbreviated as follows: G, glycine; P, proline; B, hydroxyproline; E, glutamic acid; R, arginine; K, lysine; H, histidine; F, phenylalanine; A, alanine; L, leucine; T, threonine; Y, tyrosine; εACA, ε-aminohexanoic acid-lysine-lysine-tyrosine-glycine-OH. ^b^Absorbance at 405 nm in ELISA tested with affinity-purified mAbs. In the ELISA plates, all the triple-helical peptides were coated at 4 μg/ml, whereas type II collagen (CII) was coated at 10 μg/ml. CIIC1, M2139 and UL1 are mouse monoclonal antibodies and were all tested at a concentration of 1 μg/ml; 122.9 is a rat monoclonal antibody and was tested with culture supernatant.
